# Dynamics of Toxic and Essential Element Transfer in Soil–Plant–Animal Systems Under Industrial Contamination

**DOI:** 10.3390/biology15131011

**Published:** 2026-06-25

**Authors:** Maxat Berdikulov, Karlygash Aubakirova, Olzhas Omirzakov, Vitaliy Krivets, Aigul Omarova, Almira Kuanysh, Assem Axeitova, Ali Zhanbolov, Aliya Alpamys, Madina Bralina, Maozhi Ren, Arvind Kumar Dubey, Zhadyrassyn Nurbekova

**Affiliations:** 1 National Veterinary Reference Center, Astana 010000, Kazakhstan; berdikulov.ma@mail.ru (M.B.); omirzaqov.olzhas@gmail.com (O.O.); vitaliy.krivets89@gmail.com (V.K.); aigul.s.omarova@gmail.com (A.O.); almira.kuanysh00@gmail.com (A.K.); akseitovaasem@gmail.com (A.A.); ali089684328@gmail.com (A.Z.); 2 Department of Biotechnology and Microbiology, L.N. Gumilyov Eurasian National University, Astana 010000, Kazakhstan; aubakirova_km@enu.kz (K.A.); aliya.kanatovna1819@mail.ru (A.A.); leventeem8@gmail.com (M.B.); 3 Xinjiang Key Laboratory of Functional Agriculture and Bio-Intelligent Manufacturing, Institute of Urban Agriculture, Chinese Academy of Agricultural Sciences, Chengdu 610213, China; renmaozhi01@caas.cn; 4 Department of Agronomy and Horticulture, University of Nebraska Lincoln, Lincoln, NE 68588, USA; arvindbiotech28@gmail.com

**Keywords:** toxic and essential elements, heavy metals, trace elements, soil–plant–animal system, industrial pollution, food safety, environmental contamination, transfer coefficient, inductively coupled plasma mass spectrometry

## Abstract

Industrial contamination can influence the transfer of toxic and essential elements through soil–plant–animal systems and may pose risks to food safety. This study aimed to determine whether contamination patterns in soil are reflected in forage vegetation and meat products and to evaluate trace-element behavior across interconnected components of the soil–plant–animal system. This study assessed the distribution and transfer of 12 elements (As, Be, Cd, Co, Cr, Cu, Hg, Mn, Ni, Pb, V, and Zn) in soil, forage vegetation, and meat products from five industrially affected areas of Central Kazakhstan. Element concentrations were determined by inductively coupled plasma mass spectrometry. Soil contained the highest concentrations of most elements, confirming its role as the primary reservoir of contamination, whereas forage vegetation reflected local pollution patterns. The highest levels of contamination were generally observed in the industrial centers of Temirtau and Zhezkazgan, with Zhezkazgan exhibiting the most distinct element profile. Soil-to-forage transfer was most pronounced for Cd, Cu, Pb, and Zn, with significant positive relationships between soil and forage concentrations (*p* < 0.001). Meat products generally contained lower element concentrations than soil and forage; however, Cd, Hg, and As exceeded regulatory limits in 23 of 279 samples (8.2%). By integrating environmental and animal-derived matrices within a single framework, this study provides new insight into trace-element transfer pathways and facilitates the identification of priority contaminants, high-risk areas, and livestock products requiring enhanced environmental and food safety monitoring in industrial regions.

## Simple Summary

Industrial pollution can affect agricultural ecosystems through the gradual accumulation of potentially harmful and naturally occurring elements in soil. These elements may then enter forage plants and reach animal-derived food products through animal feeding. This study examined the distribution of such elements from soil to plants and ultimately into meat products in industrial areas of Central Kazakhstan. Soil, forage, and meat samples were analyzed to understand how environmental contamination may affect food production. The highest concentrations of most elements were found in soil, confirming that soil is the main place where contamination builds up. Forage plants contained lower concentrations, although their element content reflected local pollution sources. Meat samples generally showed much lower levels than soil and forage, suggesting limited transfer into animal tissues. However, some samples exceeded food safety limits for cadmium, mercury, and arsenic. These results show the importance of monitoring soil, forage, and meat in industrial regions to assess environmental contamination and food safety risks and to support the production of safer food for local communities.

## 1. Introduction

Trace-element contamination is a key environmental and food safety concern in industrial regions. This is particularly important in soil–plant–animal systems, where elements may enter agricultural and livestock production chains. Arsenic (As), cadmium (Cd), lead (Pb), and mercury (Hg) are widely recognized as priority toxic elements and metalloids because of their environmental stability, bioaccumulative behavior, and capacity to harm biological systems. Several essential elements, including copper (Cu), zinc (Zn), manganese (Mn), cobalt (Co), and nickel (Ni), are required in trace amounts but may disrupt physiological processes at elevated concentrations. Beryllium (Be), chromium (Cr), and vanadium (V) are also relevant in multi-element monitoring because their toxic effects vary with concentration and chemical form [1,2,3]. The selection of these elements was based on their environmental and toxicological significance, as well as their association with mining, metallurgical, and processing industries that characterize the study region. These elements are among the most common industrial contaminants and can accumulate in environmental compartments and food products. Food can become an exposure route when elements from contaminated environments enter agricultural and livestock products; studies on red meat contamination indicate that animal-derived products may carry element burdens linked to local environmental contamination [4].

As long-term reservoirs, soils accumulate trace elements from natural weathering and anthropogenic sources, including atmospheric deposition, industrial dust, mining residues, and waste disposal [5,6]. In agricultural and pastoral landscapes, contaminated soil can introduce elements into a selective transfer pathway: forage plants take up the plant-available fraction, whereas only part of the absorbed load is retained in edible animal tissues [7,8,9]. Transfer is not simply proportional. Element mobility in the rhizosphere is shaped by chemical speciation, pH, redox conditions, organic matter, and microbial activity [5,6,7]. In animals, feeding conditions, absorption, and tissue-specific retention further influence element levels in meat [8,9]. For this reason, bulk soil concentrations alone cannot reliably predict levels in forage or meat; transfer factors and cross-matrix comparisons are needed to interpret movement along the chain.

In Central Kazakhstan, industrial activity has long shaped environmental conditions in urban areas and nearby pasturelands. The selected territories include Karaganda, Temirtau, Saran, Shakhtinsk, and Zhezkazgan, which differ in the dominant sources and intensity of anthropogenic impact. Temirtau is associated mainly with metallurgical production; Zhezkazgan is associated with copper mining and processing; and Karaganda, Saran, and Shakhtinsk are linked to coal extraction, power generation, and related production facilities. Previous studies have reported heavy metal enrichment and health-related risks in urban soils of Kazakhstan [10], atmospheric pollution in industrial cities of Central Kazakhstan [11], and contamination of technogenically disturbed soils in the region [12,13]. Broader regional assessments also describe sustained environmental pressure in the industrial territories of Karaganda and adjacent areas [14]. In these settings, livestock grazing on local pastures or receiving locally produced forage may be exposed to mixed trace-element contamination.

Existing literature has generally examined environmental matrices and animal-derived food products separately, rather than within a single soil–plant–animal framework. Research in the Karaganda region has documented element accumulation in soil, water, and vegetation in industrial areas [15], whereas recent work in Kazakhstan has examined trace-element contamination in animal-derived foods [16]. These findings provide useful reference points, but they do not jointly assess soil contamination, forage uptake, and element levels in meat. Thus, it remains unclear how environmental contamination in the study areas relates to element levels in forage vegetation and locally produced meat.

Accordingly, this study aimed to assess whether territorial patterns of toxic and essential elements in soil are also reflected in forage vegetation and meat products from industrially affected areas of Central Kazakhstan. Samples from five study areas were analyzed for twelve elements—As, Be, Cd, Co, Cr, Cu, Hg, Mn, Ni, Pb, V, and Zn—using inductively coupled plasma mass spectrometry (ICP-MS). The analysis focused on three related aspects: differences in element accumulation among territories, soil-to-forage transfer, and associations between forage element profiles and those in beef, horse meat, and mutton collected from the same territories. The scientific objective was to evaluate the behavior of trace elements across interconnected components of the regional soil–plant–animal system and to determine whether contamination patterns observed in environmental matrices are reflected in animal-derived food products. By integrating soil, forage vegetation, and meat products within a single soil–plant–animal framework, this study extends previous regional assessments that considered environmental contamination and animal-derived food products separately. This integrated approach provides new insight into element transfer pathways in industrial regions and helps identify priority elements, affected areas, and livestock products requiring enhanced monitoring and food safety assessment.

## 2. Materials and Methods

### 2.1. Study Area

The study was conducted in Central Kazakhstan, within the Karaganda and Ulytau regions, near the industrial cities of Karaganda, Temirtau, Saran, Shakhtinsk, and Zhezkazgan. These territories are characterized by intensive industrial activity, including mining, coal extraction and processing, metallurgy, energy production, and construction-material industries. Major industrial facilities include coal mines, coal-processing plants, cement factories, thermal power plants, tailing storage facilities, and other industrial enterprises that may contribute to environmental contamination. These territories were selected because of their high environmental burden and the presence of major industrial facilities that may release toxic and essential elements into the environment.

**Figure 1:**
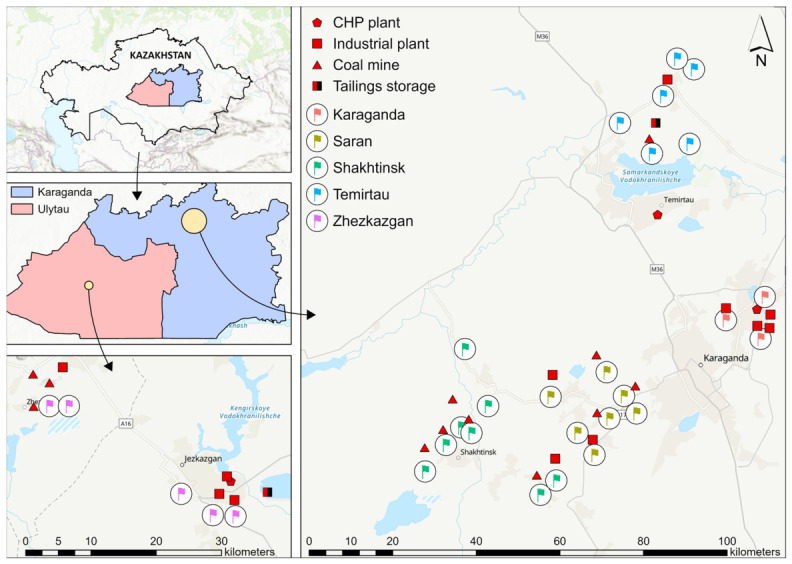
Location Map of the study areas and sampling locations. The Karaganda Region is shown in blue, and the Ulytau Region is shown in pink. Yellow circles indicate the study areas surrounding the major industrial centers. Colored flags mark the sampling locations in Karaganda, Temirtau, Saran, Shakhtinsk, and Zhezkazgan. Symbols indicate the major industrial facilities. The map was produced using ArcGIS Pro 3.5.0 (Esri, Redlands, CA, USA).

The soils of Karaganda and the surrounding areas are mainly represented by chestnut soils and their subtypes. Pasture vegetation in these territories is dominated by grass and wormwood communities. In Zhezkazgan and the nearby city of Satpayev, soils are mainly brown, gray-brown, and saline. Local pasture vegetation is represented by wormwood, ephemeral plants, fescue, and cereal grasses. The location of the studied cities, sampling sites, and main industrial facilities considered as potential sources of element release into the environment is shown in Figure 1.

### 2.2. Sampling Procedure and Initial Processing

Soil samples were collected near facilities considered potential sources of heavy metal emissions at a distance of 1–3 km. This distance range was selected considering the size of the sanitary protection zones surrounding industrial enterprises, the distribution of pasture lands, and the landscape characteristics of the study area. Sampling directions were selected using wind rose diagrams based on data from the nearest meteorological station for the previous year. The sampling sites were chosen to correspond to livestock pasture areas. Each site was divided into 6–12 test plots of 1.5 ha. Within each plot, five subsamples were collected at 10 m intervals using the envelope method from a depth of 10–20 cm. The slight variation in sampling depth reflected local site conditions; however, in all cases, the upper root zone of the soil was targeted. This depth interval corresponds to the main root zone of forage vegetation and represents the soil layer most directly influenced by atmospheric deposition and anthropogenic contamination, making it the most relevant layer for assessing element transfer to plants. The subsamples were mixed to obtain a composite sample weighing approximately 1 kg. Soil samples were collected using a soil sampler GeoSampler (Bürkle GmbH, Bad Bellingen, Germany), placed in cotton bags, and labeled. After delivery to the laboratory, the samples were air-dried, spread on paper, ground with a pestle, and passed through a 2 mm sieve to remove stones, plant roots, glass fragments, and other debris. The sieved sample was quartered by placing it on paper in a square shape, dividing it into four equal parts, and combining two opposite quarters. This procedure was repeated until a representative sample of approximately 50 g was obtained. In total, 203 soil samples were collected.

Forage samples were collected from the same plots and test sites as the soil samples. The forage sampling points corresponded to the soil sampling points. All forage grasses and grass mixtures typical of each territory were collected without separation by species. The aboveground forage material was cut approximately 3–5 cm above the soil surface. Ceramic scissors and knives were used for sampling, and the samples were placed in cotton bags. Five subsamples, each weighing up to 200 g, were collected from each test site using the envelope method at 10 m intervals. The material was then placed on a polyethylene sheet, thoroughly mixed, and combined into a composite sample. From 10 different parts of the composite material, 50–100 g portions were taken to obtain a representative sample weighing 0.5–1 kg. After delivery to the laboratory, the samples were air-dried, crushed using a shredder POWTEQ Knife Mill HM100 (Beijing Grinder Instrument Co., Ltd., Beijing, China), mixed, and reduced to an analytical sample of approximately 50 g. In total, 203 forage samples were collected.

Raw meat samples, including beef, horse meat, and mutton, were collected from slaughterhouses, markets, and agricultural fairs in the studied cities. When selecting meat samples, only products originating from the studied administrative territories were included. The origin of the meat was verified to ensure correspondence with the same areas where soil and forage samples were collected. The samples were placed in zip-lock plastic bags and transported to the laboratory in insulated containers with cooling elements under controlled temperature conditions. In the laboratory, meat samples were homogenized and stored in a freezer until analysis. In total, 193 beef samples, 44 horse meat samples, and 42 mutton samples were collected [17,18,19,20].

### 2.3. Sample Preparation

Samples were prepared by acid mineralization under elevated pressure using a laboratory microwave digestion system (Multiwave GO Plus System, Anton Paar GmbH, Graz, Austria). Soil, forage, and meat samples were digested according to standard wet decomposition protocols. Before digestion, all samples were weighed using an analytical balance (ATX224, Shimadzu, Kyoto, Japan).

For soil samples, 0.3 g of material was digested with 8 mL of concentrated nitric acid (Thermo Scientific Chemicals, Kandel, Germany) and 3 mL of hydrogen peroxide (JSC ECOS-1, Staraya Kupavna, Moscow Region, Russia). After mineralization, the digest was transferred into 50 mL polypropylene tubes and filtered through PTFE membrane filters with a pore size of 0.45 µm (Agilent Technologies, Santa Clara, CA, USA). The filtrate was then diluted 11-fold with deionized water.

For forage samples, 0.2 g of material was digested with 6 mL of concentrated nitric acid and 1 mL of hydrogen peroxide. After mineralization, the digest was transferred into 50 mL polypropylene tubes and filtered through PTFE membrane filters with a pore size of 0.44 µm. The filtrate was then diluted 8-fold with deionized water.

For meat samples, 0.4 g of homogenized material was digested with 6 mL of concentrated nitric acid. After mineralization, the digest was transferred into 50 mL polypropylene tubes and diluted 3-fold with deionized water.

The mass fraction of elements was calculated using the sample mass, the final solution volume after mineralization, and the dilution factor according to the following equation:

Element(C) = (*Df* × *ICP*(*C*) × *V*)/*m*, (1)

where Element(C) is the concentration of the element in the sample, mg/kg; *Df* is the dilution factor; *ICP*(*C*) is the element concentration measured by ICP-MS, mg/L; *V* is the final solution volume, L; and *m* is the raw sample mass, kg.

For each analytical series, a blank control sample was prepared and processed through all stages of sample preparation under the same conditions as the main samples, but without the test material. Element concentrations detected in the blank samples were subtracted from the measured concentrations of the studied samples [21,22].

### 2.4. Element Analysis

The elemental composition of the samples was determined by inductively coupled plasma mass spectrometry (ICP-MS) using an Agilent 7700 system (Agilent Technologies, Santa Clara, CA, USA) controlled by MassHunter software version A.01.02 (Agilent Technologies, Santa Clara, CA, USA). For data verification and quality control in cases of atypical results, an Agilent 7500 ICP-MS system was additionally used.

For each sample, the measurement procedure included a single sample introduction followed by three spectral scans within one analytical cycle. The results of sequential scans were automatically averaged, which supported the accuracy and reproducibility of the measurements without additional independent analytical replicates. Calibration curves were constructed for each measurement series and showed strong linearity, with correlation coefficients of approximately 0.999.

Working calibration solutions at concentrations of 0, 5, 10, 20, 50, and 100 µg/L were prepared in deionized water by sequential dilution of the multi-element standard IV ICPMS-71A (Inorganic Ventures, Christiansburg, VA, USA) and the single-element mercury standard MSHGN-10PPM (Inorganic Ventures, Christiansburg, VA, USA). Measurements were performed in ascending order of concentration. When the concentration of an element exceeded the upper calibration limit, the sample was additionally diluted and reanalyzed within the working calibration range to ensure correct quantitative determination.

### 2.5. Statistical Analysis

The normality of data distribution was assessed using the Shapiro–Wilk test. Because the element concentration data did not always meet the assumptions of normal distribution, differences among territories and sample types were analyzed using the nonparametric Kruskal–Wallis test. When statistically significant differences were detected, post hoc pairwise comparisons were performed using Dunn’s test with Bonferroni correction to reduce the risk of errors related to multiple comparisons. The threshold for statistical significance was set at *p* < 0.05.

To assess the transfer of elements from soil to forage, transfer coefficients were calculated as the ratio of element concentration in forage to its concentration in soil. Hierarchical cluster analysis was performed using standardized mean element concentrations, Euclidean distance, and the Ward.D2 method. Principal component analysis was used to evaluate the structure of variability in the elemental composition of soil, forage, and meat samples. Spearman’s rank correlation analysis was applied to assess associations between element concentrations in forage and meat samples.

All calculations and statistical analyses were performed in the R software environment, version 4.5.1.

## 3. Results

### 3.1. General Characteristics of Element Distribution

**Figure 2:**
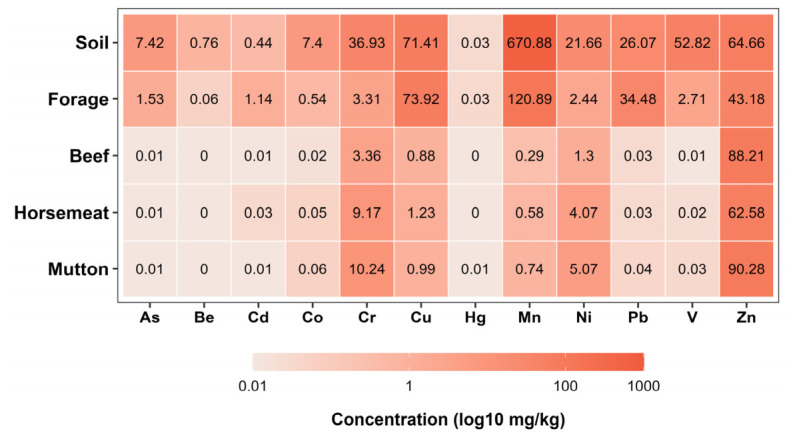
Mean concentrations of the studied elements in soil, forage vegetation, and meat products. Numerical values within the cells indicate mean element concentrations (mg/kg), whereas color intensity reflects their relative magnitude on a logarithmic scale, with darker shades corresponding to higher concentrations.

The analysis of average element concentrations showed clear differences among soil, forage, and meat samples (Figure 2). The highest concentrations of most elements were recorded in soil. Meat samples are presented separately by animal species, including beef, horse meat, and mutton. In the following multivariate analyses, these samples were combined into a single meat group to simplify the assessment of territorial differences.

Soil samples generally exhibited the highest concentrations of the studied elements (Figure 2). For most studied elements, concentration in soil differed significantly from that in forage and meat samples, particularly from meat products (*p* < 0.05).

Forage samples occupied an intermediate position between soil and meat products. For several elements, forage concentrations were comparable to or close to soil values. This pattern was most pronounced for Cu, with mean concentrations of 73.92 mg/kg in forage and 71.41 mg/kg in soil, although the difference between these matrices remained statistically significant (*p* < 0.001). Relatively high concentrations of Mn (120.89 mg/kg), Zn (43.18 mg/kg), and Pb (34.48 mg/kg) were also observed in forage.

Specific distribution patterns were observed for Cd and Hg. The mean Cd concentration in forage was 1.14 mg/kg, which was higher than in soil (0.44 mg/kg), although the difference between forage and soil was not statistically significant (*p* = 0.246). For Hg, the difference between forage and soil was also not statistically significant (*p* = 1.0), with similar mean concentrations of 0.03 mg/kg in both matrices.

Meat products were characterized by substantially lower concentrations of most elements compared with soil and forage. This was confirmed by statistically significant differences in most soil–meat and forage–meat comparisons (*p* < 0.05). At the same time, several elements had comparatively high concentrations in animal tissues. Zinc was the most abundant element in all meat types, although its concentrations varied among animal species (Figure 2). No statistically significant differences in Zn were found between beef and mutton (*p* = 0.693), whereas horse meat differed from mutton (*p* = 0.006).

Elevated Cr concentrations were observed in mutton (10.24 mg/kg) and horse meat (9.17 mg/kg), which were significantly higher than in beef (*p* < 0.001). Nickel concentrations were generally higher in mutton and horse meat than in beef (Figure 2).

The concentrations of As, Pb, and Hg in meat products remained low and showed no clear differences between meat types. In most cases, no statistically significant differences were found between beef, horse meat, and mutton for these elements (*p* > 0.05).

In general, the distribution pattern showed a decrease in element concentrations from soil and forage to meat products. However, several elements, particularly Zn, Cr, Ni, and Cd, had comparatively high values in specific components of the studied system.

### 3.2. Spatial Variability of Elements in Soil, Forage, and Meat Samples

The average concentrations of the studied elements differed among both sample types and the studied territories (Table 1). The highest levels of most elements were recorded in soil samples. Element concentrations were generally lower in forage vegetation, although clear spatial differences remained. In meat products, most elements were detected at lower levels, with the exception of Zn, which remained relatively high across all studied areas.

Soil. In soil samples, the most pronounced spatial differences were observed for Cu, Pb, As, Cd, Zn, and Mn. For As, the lowest mean concentrations were found in Karaganda, Saran, and Shakhtinsk, ranging from 5.51 to 5.70 mg/kg. Higher values were recorded in Temirtau and Zhezkazgan, where As concentrations reached 9.16 and 12.37 mg/kg, respectively. Statistically significant differences were found between Temirtau and the three territories with lower As levels, as well as between Zhezkazgan and Karaganda, Saran, and Shakhtinsk (*p* < 0.001).

For Cd, a similar spatial trend was observed, with elevated concentrations in Temirtau and the highest levels in Zhezkazgan (Table 1). Both territories differed significantly from those with lower Cd concentrations (*p* < 0.01). Cu showed the strongest territorial contrast, with markedly elevated concentrations in Zhezkazgan compared with the other territories (Table 1). Statistically significant differences were confirmed between Zhezkazgan and Karaganda, Saran, and Shakhtinsk (*p* < 0.001), while the difference between Zhezkazgan and Temirtau was not statistically significant (*p* = 0.192).

A similar pattern was observed for Pb. Karaganda, Saran, and Shakhtinsk showed relatively lower Pb levels, whereas elevated concentrations were observed in Temirtau and especially in Zhezkazgan (Table 1). Statistically significant differences were found between Temirtau and the territories with lower Pb levels (*p* < 0.01), as well as between Zhezkazgan and Karaganda, Saran, and Shakhtinsk (*p* < 0.001).

Zn and Mn were also characterized by elevated concentrations in Temirtau and Zhezkazgan, whereas the lowest values were more often recorded in Saran. For Be, territorial differences were moderate: the lowest concentration was observed in Saran (0.58 mg/kg), while higher values were recorded in Temirtau (0.84 mg/kg) and Zhezkazgan (1.04 mg/kg). Significant differences were confirmed between Saran and Temirtau (*p* = 0.001), as well as between Saran and Zhezkazgan (*p* < 0.05).

For V and Cr, Saran also showed the lowest concentrations. V reached 41.10 mg/kg in Saran, while higher values were found in Karaganda (60.52 mg/kg), Temirtau (58.41 mg/kg), and Zhezkazgan (68.05 mg/kg). Statistically significant differences were found between Saran and the territories of Karaganda, Temirtau, and Zhezkazgan (*p* < 0.001).

For Cr, the lowest level was recorded in Saran (26.63 mg/kg), whereas Temirtau and Zhezkazgan showed higher concentrations of 43.99 and 50.72 mg/kg, respectively. Significant differences were found between Saran and the territories with higher V and Cr levels (*p* < 0.05).

For Co, territorial differences were less pronounced, although Shakhtinsk showed significantly lower values than Temirtau (*p* < 0.001). For Ni, Saran showed the lowest levels, whereas Zhezkazgan exhibited the highest levels among the studied territories (Table 1). Significant differences were confirmed between Saran and Temirtau (*p* < 0.001) and between Saran and Zhezkazgan (*p* < 0.001). Hg concentrations remained low in all territories, ranging from 0.02 to 0.04 mg/kg, with a significantly lower level in Shakhtinsk compared with Karaganda (*p* = 0.008) and Temirtau (*p* = 0.004).

Forage. Spatial heterogeneity was also observed in forage vegetation, although the range of differences was generally less pronounced than in soil. For As, concentrations were lowest in Karaganda and Saran, intermediate in Shakhtinsk and Temirtau, and highest in Zhezkazgan (Table 1). Zhezkazgan differed significantly from all other territories (*p* < 0.01).

For Cd, concentrations were generally similar among Karaganda, Saran, Shakhtinsk, and Temirtau, whereas substantially higher levels were observed in Zhezkazgan (Table 1). This difference was statistically significant (*p* < 0.001). Cu showed a similar territorial pattern, with concentrations increasing from Karaganda and Saran toward Shakhtinsk, Temirtau and Zhezkazgan (Table 1). The differences between Temirtau and the territories with lower Cu levels were statistically significant, while Zhezkazgan significantly exceeded all territories (*p* < 0.01) except Temirtau (*p* = 0.129).

For Pb, the lowest concentration was observed in Saran, intermediate in Karaganda and Temirtau, and highest in Zhezkazgan (Table 1). Zhezkazgan showed significantly higher Pb levels than all other territories (*p* < 0.01). For Be, concentrations were lower in Karaganda and Saran and higher in Shakhtinsk, Temirtau, and Zhezkazgan (Table 1). These differences were statistically significant (*p* < 0.001).

For V and Cr, the lowest concentrations were observed in Saran, whereas higher levels were in Shakhtinsk, Temirtau, and Zhezkazgan (Table 1). Significant differences were found between Saran and the territories with higher V and Cr concentrations (*p* < 0.05).

For Co, lower values were observed in Karaganda and Saran, while higher levels were recorded in Temirtau and Zhezkazgan (Table 1). For Ni, the lowest concentration was observed in Saran, whereas the highest values were recorded in Zhezkazgan (*p* < 0.001) (Table 1). Hg concentrations remained low in all forage samples, although significantly higher levels were observed in comparison with Karaganda and Saran (*p* < 0.001).

Forage vegetation showed clear territorial differences, with the highest concentrations of several elements recorded in Zhezkazgan. Detailed descriptive statistics, including the mean, median, standard deviation, minimum, maximum, and sample size values for all studied elements and matrices, are provided in Supplementary Table S1. The elevated Cu and Pb concentrations observed in forage samples from Zhezkazgan were confirmed by quality-controlled ICP-MS analyses and are therefore interpreted as reflecting local contamination rather than analytical artifacts.

Meat products. In meat products, spatial differences were less pronounced than in soil and forage, but they remained evident for individual elements. For As, Cd, and Pb, concentrations were low in most areas, although the highest mean values were recorded in Zhezkazgan, where differences from several other territories were statistically significant.

In the studied meat samples, exceedances of the As regulatory limit were detected only in one beef sample from Temirtau, where the concentration reached 0.14 mg/kg against the established limit of 0.10 mg/kg under the Technical Regulations of the Eurasian Economic Union. Detailed information on the regulatory limits established by the Technical Regulations of the Eurasian Economic Union (EAEU) and the frequency of exceedances for Cd, Hg, and As in different meat types is presented in Table 2. Overall, exceedances were most frequently observed for Cd, followed by Hg, whereas As exceeded the regulatory limit in only one sample.

For Cd, the largest number of samples exceeding the established limit was detected at the regulatory value of 0.05 mg/kg. Exceedances were found in one beef sample from Temirtau and four beef samples from Zhezkazgan. Among horse meat samples, exceedances were detected in one sample from Shakhtinsk, four samples from Temirtau, one sample from Saran, one sample from Karaganda, and two samples from Zhezkazgan. In addition, one mutton sample from Zhezkazgan exceeded the Cd limit. No Pb exceedances were detected in the studied meat samples.

For Hg, concentrations remained low in all territories, and a statistically significant difference was found only between Temirtau and Zhezkazgan (*p* = 0.04). However, nonconformity with the Hg limit established by the same regulation was detected in individual beef samples from Karaganda, Saran, and Temirtau. Hg exceedances were also recorded in mutton samples: one sample from Karaganda and three samples from Zhezkazgan.

Exceedances for As, Cd, and Hg were detected in 23 of the 279 studied meat samples, corresponding to approximately 8%. Cd was the most frequently exceeded element, especially in samples from Zhezkazgan and Temirtau. By product type, the highest frequency of nonconforming samples was observed in horse meat, followed by beef and mutton. Exceedances in horse meat were registered across all studied cities.

For Be and V, concentrations remained low in all territories, although Zhezkazgan showed significantly higher V levels than Karaganda, Saran, and Temirtau (*p* < 0.05). Cr showed a clearer territorial pattern, with lower concentrations in Karaganda and Temirtau and higher values in Saran, Shakhtinsk, and Zhezkazgan (Table 1). Significant differences were found between Shakhtinsk and Karaganda (*p* = 0.003), as well as between Zhezkazgan and several other territories (*p* < 0.001), except Shakhtinsk.

For Co, concentrations generally increased toward Zhezkazgan, which showed significantly higher levels than the other territories (*p* < 0.001), except Shakhtinsk (Table 1). For Ni, concentrations were lowest in Karaganda and highest in Zhezkazgan (Table 1). Significant differences were found between Zhezkazgan and Karaganda, Saran, and Temirtau (*p* < 0.001). Mn was lower in Karaganda and Temirtau and higher in Shakhtinsk and Zhezkazgan, with significant differences mainly between Zhezkazgan and territories with minimal Mn levels (*p* < 0.001).

Cu concentrations were lowest in Karaganda and Temirtau, while the highest values were recorded in Zhezkazgan. Significant differences were found mainly between Zhezkazgan and territories with lower Cu levels (*p* < 0.05), except Shakhtinsk. Zn remained relatively high across all territories, although its spatial variability was less pronounced than that of several other elements. Nevertheless, differences between areas with lower and higher Zn levels were still observed.

Meat products, therefore, showed lower element concentrations than soil and forage, but territorial differences remained visible for several elements, especially in Zhezkazgan.

### 3.3. Multivariate Assessment of Territorial Differences

**Figure 3:**
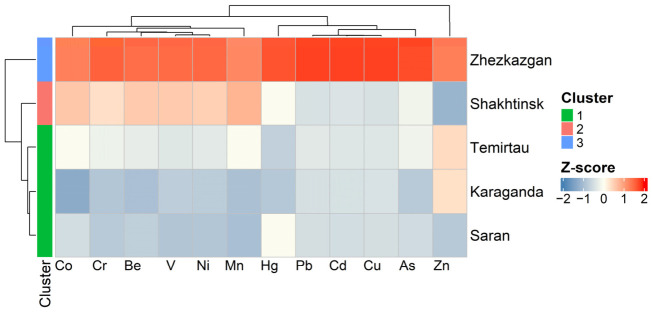
Heatmap and hierarchical clustering dendrogram of the studied territories based on standardized mean concentrations of the analyzed elements. The color scale represents standardized values (Z-scores), where positive values indicate concentrations above the overall mean and negative values indicate concentrations below the overall mean. Territories with similar elemental composition are grouped according to hierarchical cluster analysis.

Hierarchical cluster analysis was used to evaluate territorial differences in the elemental composition of the studied samples. The analysis was performed using standardized mean element concentrations, Euclidean distance, and the Ward.D2 linkage method (Figure 3). The dendrogram separated the studied territories into three clusters. Zhezkazgan formed a separate cluster and had the most distinct elemental profile among the studied sites. Shakhtinsk formed an intermediate cluster, whereas Karaganda, Saran, and Temirtau were grouped together, showing closer similarity in their average elemental composition.

The heat map showed that Zhezkazgan had the highest standardized values for most elements, especially Cu, Pb, Cd, As, and Zn. This corresponds to the elevated concentrations previously observed in soil, forage, and meat samples. Shakhtinsk showed a moderate increase in several elements, while Karaganda, Saran, and Temirtau had a more balanced distribution profile.

The element dendrogram also showed grouping based on similarity in spatial distribution. Pb, Cd, and Cu formed a closely related cluster, suggesting similar distribution patterns. As and Hg joined this group at the next level of similarity. A separate group included Co, Mn, Ni, Be, V, and Cr, which showed a more consistent pattern of variation across territories. Zn occupied an intermediate position between the main clusters.

Cluster analysis confirmed territorial heterogeneity in the elemental composition of the studied samples and complemented the results of comparative statistical analysis.

**Figure 4:**
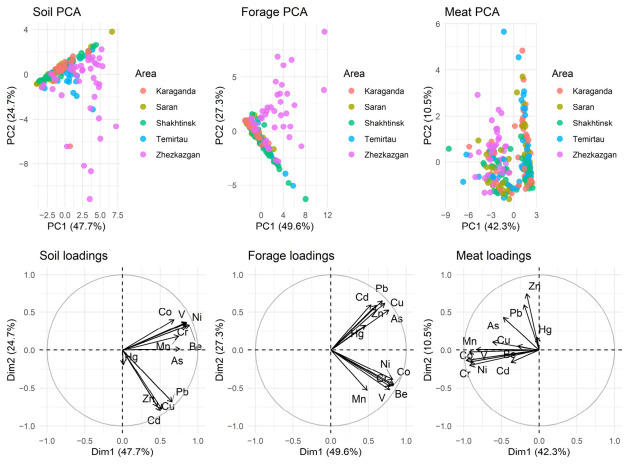
Principal component analysis (PCA) of the elemental composition of soil, forage vegetation, and meat samples. PC1 and PC2 represent the first and second principal components, respectively. The percentages shown in parentheses on the axes indicate the proportion of the total variance explained by each principal component, where the total variance of all analyzed elements is considered 100%. In the loading plots, Dim1 and Dim2 correspond to the first (PC1) and second (PC2) principal components, respectively.

Principal component analysis (PCA) was then used to assess the structure of variability in soil, forage, and meat products (Figure 4). A detailed description of the PCA methodology, including the calculation of principal components and the interpretation of PC1, PC2, Dim1, and Dim2, is provided in Supplementary Material S2. PCA was performed separately for each sample type using standardized individual element concentrations. Biplots were used to interpret the contribution of individual elements to the separation of samples.

In soil samples, the first two principal components explained 72.4% of the total variability, with PC1 accounting for 47.7% and PC2 for 24.7%. The PCA plot showed a clear separation of part of the Zhezkazgan samples, which corresponds to the elevated concentrations of several elements observed in this territory. According to the loading plot, Cu, Cd, Pb, and Zn made the greatest contribution to soil sample differentiation, with vectors directed toward the same region of the factor space. This suggests a similar pattern of spatial distribution for these elements. Co, V, and Ni also contributed to the separation of soil samples, but their contribution was less pronounced.

For forage samples, the first two components explained 76.9% of the total variability, with PC1 accounting for 49.6% and PC2 for 27.3%. As in soil, part of the Zhezkazgan samples remained separated from the other territories, although the overall contrast was less pronounced. Pb, Cd, Cu, and As contributed most strongly to the separation of forage samples, which agrees with the differences in mean concentrations described above. At the same time, Mn, Co, Ni, and Be formed a separate direction of variability, suggesting a more complex distribution of elements in forage samples.

In meat products, the first two components explained 52.8% of the total variability, with PC1 accounting for 42.3% and PC2 for 10.5%. This value was lower than in soil and forage samples. The PCA plot showed greater overlap between samples from different territories, indicating weaker territorial differentiation in meat products. According to the loading plot, Zn, Pb, and Hg contributed most to the variability of meat samples, while most other elements were grouped closer together. The territorial signal was strongest in soil, remained visible in forage, and became weaker in meat products.

### 3.4. Soil-to-Forage Transfer of Elements

**Figure 5:**
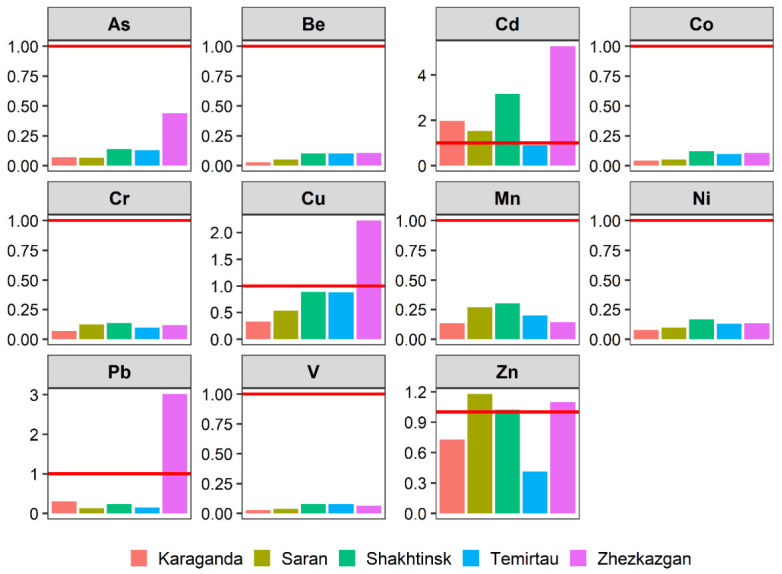
Average transfer coefficients of chemical elements in the soil–forage system across the studied territories. The red horizontal line indicates TF = 1, where element concentrations in forage and soil are equal; values above this level indicate more pronounced transfer from soil to forage vegetation. Transfer factor (TF) values were calculated as the ratio of the mean concentration of each element in forage vegetation to its corresponding mean concentration in soil within each studied territory.

To further assess element migration in the soil–forage system, transfer coefficients were calculated as the ratio of element concentration in forage to its concentration in soil for each studied territory (Figure 5). For most elements, TF values remained below 1, indicating limited transfer from soil to forage vegetation.

The highest TF values were observed for Cd, Cu, and Pb. Cd was the most notable element, with TF values exceeding 1 in all studied territories and reaching the highest level in Zhezkazgan. This indicates a relatively high transfer of Cd into forage vegetation compared with the other studied elements.

A similar pattern was observed for Cu. In Zhezkazgan, Cu showed TF values above 1 and clearly exceeded the values recorded in other territories. This result is consistent with the high Cu concentrations observed in both soil and forage samples from this area. For Pb, TF exceeded 1 only in Zhezkazgan, while in other territories the values remained substantially lower. It should be noted that the calculated TF values were based on the ratios of mean element concentrations in forage vegetation and soils for each studied territory rather than on paired samples collected from the same sampling locations. Therefore, the obtained coefficients reflect general territorial trends in element transfer and should not be interpreted as direct soil–forage relationships at the individual sampling-point level.

In addition, the bioavailability and plant uptake of elements may be strongly influenced by soil properties such as pH, organic matter content, and element speciation. As these parameters were not determined in the present study, the interpretation of TF values, particularly for Cd, should be considered with caution, given its relatively high mobility in the soil–plant system.

For Zn, TF values in Saran, Shakhtinsk, and Zhezkazgan approached or slightly exceeded 1, indicating relatively stable transfer of this element into forage vegetation.

For As, Be, Co, Cr, Mn, Ni, and V, transfer coefficients remained low in all territories. This indicates weaker accumulation of these elements in forage biomass and limited transfer within the soil–forage system. Hg was excluded from the visualization of transfer coefficients because soil concentrations were often close to zero, which would produce unstable and overestimated TF values.

**Figure 6:**
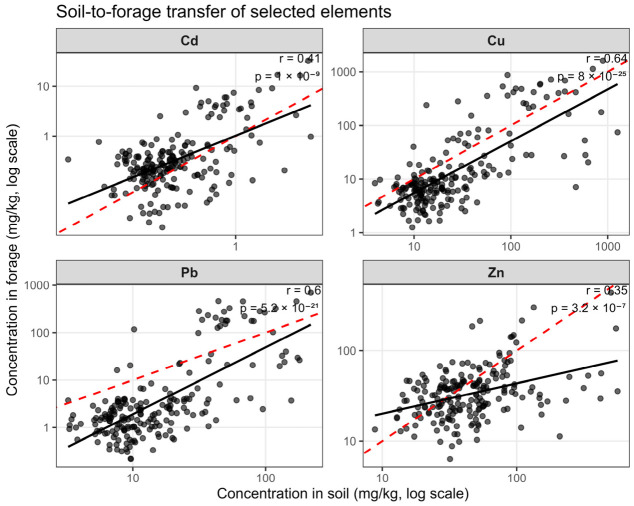
Relationships between Cu, Pb, Cd, and Zn concentrations in soil and forage vegetation. Axes are presented on a logarithmic scale (log10). Black solid lines indicate linear trend lines, whereas the red dashed line represents the 1:1 relationship between element concentrations in soil and forage vegetation. Each point corresponds to an individual sample. Values of r and *p* represent Spearman’s correlation coefficient and its significance level, respectively.

The higher TF values observed for Cd, Cu, Pb, and Zn were further supported by the relationships between their concentrations in soil and forage samples (Figure 6). Positive and statistically significant correlations were found for these elements.

The strongest relationships were observed for Cu (r = 0.64; *p* < 0.001) and Pb (r = 0.60; *p* < 0.001). In both cases, higher soil concentrations were associated with higher concentrations in forage. For Cd, the relationship was moderate (r = 0.41; *p* < 0.001), although this element showed the highest TF values.

A significant positive relationship was also found for Zn (r = 0.35; *p* < 0.001), but it was weaker than for Cu and Pb. Thus, Cd, Cu, Pb, and Zn showed the most pronounced soil-to-forage transfer among the studied elements.

### 3.5. Associations Between Forage and Meat Element Profiles

**Figure 7:**
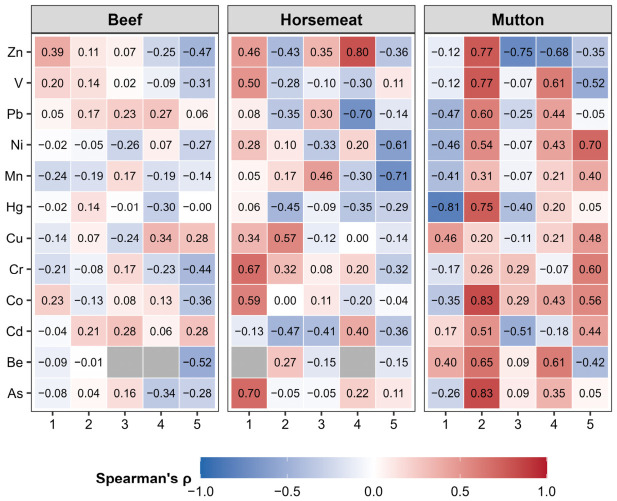
Spearman’s rank correlation coefficients (ρ) describing the relationships between element concentrations in forage vegetation and different meat types across the studied territories: 1—Karaganda, 2—Saran, 3—Shakhtinsk, 4—Temirtau, and 5—Zhezkazgan. The color scale indicates the direction and strength of the correlation, with positive correlations shown in red and negative correlations shown in blue. Gray cells indicate cases where the correlation coefficient could not be calculated due to a lack of data variation.

Since spatially matched forage–meat pairs were not available at the level of individual sampling points, forage–meat relationships were assessed using Spearman’s rank correlation analysis at the territorial level (Figure 7). The analysis was performed separately for each studied territory, meat type, and element. This approach was used to compare element-specific associations between forage vegetation and animal products without assuming direct point-to-point transfer. A detailed description of Spearman’s rank correlation analysis, including the equation used for calculating the correlation coefficient, is provided in Supplementary Material S3.

Spearman’s correlation analysis showed that forage–meat associations depended on both meat type and territory. The strongest positive correlations were observed mainly for mutton, especially in Saran, whereas beef generally showed weak or near-zero correlations. Horse meat showed an intermediate and more territory-dependent pattern.

For beef, correlations between forage and meat element concentrations were generally weak across most elements.

Even for elements with relatively high soil-to-forage transfer, such as Cd, Cu, Pb, and Zn, stable forage–meat correlations were not observed across territories. Only limited positive associations were observed for selected elements and territories, indicating generally weak forage–meat relationships in beef.

Horse meat showed stronger territorial heterogeneity, with several moderate to strong positive associations observed for selected elements across different territories. Negative associations were also observed for several elements, indicating that forage–meat relationships varied considerably among territories (Figure 7). At the same time, negative correlations were observed for several elements in specific territories (Figure 7).

The strongest agreement between forage and meat profiles was observed for mutton. Strong positive forage–meat correlations were particularly evident in Saran, while positive associations were also observed in Temirtau and Zhezkazgan (Figure 7).

Among individual elements, Zn showed high positive correlations in selected cases, particularly in mutton from Saran and horse meat from Temirtau, but this association was not consistent across all territories. Cr showed positive correlations mainly in horse meat and mutton, although its pattern also varied by territory. Cu showed moderate positive associations in horse meat and mutton, while correlations in beef remained weak. Cd and Pb were more variable: positive associations were mainly observed in Saran, Temirtau, and Zhezkazgan but were not evident in beef. For Be and As, associations in beef and partly in horse meat remained weak or unstable.

Overall, forage–meat associations were stronger in mutton and, to a lesser extent, in horse meat than in beef. Geographically, the strongest forage–meat similarity was observed in Saran, especially for mutton, whereas Zhezkazgan showed a more heterogeneous pattern with both positive and negative correlations.

## 4. Discussion

The present study showed a clear matrix-dependent and territorial pattern in the distribution of toxic and essential elements within the soil–forage–meat system. Soil contained the highest concentrations of most elements, while forage vegetation had lower values but still retained territorial differences. Meat products generally showed much lower element concentrations than soil and forage; however, exceedances of regulatory limits for Cd, Hg, and As indicate that food safety risks cannot be excluded. The soil-to-forage transfer assessment identified Cd, Cu, Pb, and Zn as the elements with the most pronounced transfer into forage vegetation. Forage–meat associations were not uniform and depended on both territory and meat type, with stronger correlations observed for mutton and, to a lesser extent, horse meat than for beef.

Soil had the highest concentrations of most studied elements, which is consistent with the general behavior of trace elements in terrestrial ecosystems. Soils can retain potentially toxic elements for a long time because many of them are persistent and only slowly removed from the upper soil layer. Their mobility and availability to plants depend on soil properties, element form, organic matter, pH, and other geochemical conditions [5,6]. The clear separation of Zhezkazgan in the cluster analysis and PCA agrees with previous reports showing that industrial and mining areas of Kazakhstan are often characterized by elevated concentrations of Cd, Cu, Pb, Zn, and other trace elements in soils and plants [10,12,13,14,15,23,24]. Similar patterns have also been reported for mining-affected areas in Central Asia, where contamination was observed not only in environmental samples but also in grazing livestock [25]. In the present study, Zhezkazgan and, for some elements, Temirtau showed higher concentrations than the other territories, but these results should be interpreted as territorial contamination patterns rather than direct evidence of specific emission sources.

Forage vegetation occupied an intermediate position between soil and meat products, but it did not simply repeat the soil profile. This is expected because plant uptake depends not only on total soil concentration but also on element mobility, bioavailability, soil properties, and plant-specific accumulation capacity [5,6,8]. In the present study, Cd, Cu, Pb, and Zn showed the most pronounced soil-to-forage transfer. Differences between the observed regression trends and the 1:1 reference relationship indicate that increasing concentrations in soil were not always accompanied by proportional increases in forage concentrations. For Cd, Cu, and Pb, the regression trends generally followed the direction of the 1:1 line, suggesting relatively consistent increases in element concentrations within the soil–forage system. In contrast, Zn showed the most pronounced deviation, with concentrations in forage increasing much more slowly than those in soil. This behavior may reflect the essential role of Zn and physiological mechanisms regulating its uptake and accumulation in plants. Consequently, even under elevated soil Zn concentrations, transfer to aboveground biomass may not occur proportionally.

Similar patterns have been reported in studies on wild grass, pasture vegetation, and forage plants from industrial, mining, and smelting areas, where transfer and accumulation were strongly element-specific [26,27,28,29,30,31,32]. The relatively high transfer of Cd is especially important because this element can be taken up by plants even when its total concentration in soil is not the highest among the measured elements [5,6,27,28]. At the same time, the weaker transfer of As, Cr, Ni, Mn, Co, and V in our study suggests that their movement into forage vegetation was more limited under the studied conditions.

Meat products contained lower concentrations of most elements than soil and forage, which indicates that element transfer into animal tissues was limited. This pattern is consistent with studies showing that the movement of metals from feed and the environment into edible animal tissues depends on absorption, metabolism, tissue distribution, and excretion processes [9,33,34]. However, lower concentrations in meat do not mean the absence of risk. In the present study, exceedances were detected for Cd, Hg, and As, which are among the most important toxic elements in food safety assessment [1,2,3,4,35,36,37,38,39]. Previous studies on meat and animal-derived food products have shown that Cd, Pb, Hg, As, Cr, and Ni may occur at variable levels depending on environmental exposure, animal species, feeding conditions, and regional contamination background [35,36,37,38,39,40,41]. Therefore, the detected exceedances, even if limited to a relatively small proportion of samples, show that meat products from industrially affected territories require regular monitoring.

The stronger forage–meat associations observed for mutton and horse meat may be related to differences in animal management and exposure. In grazing-based systems, animals can receive metals not only from forage but also from soil particles, dust, water, and other environmental sources [25,42,43]. Previous studies have shown that metal accumulation in livestock depends on pasture contamination, feed composition, animal species, age, and exposure duration [40,42,43,44,45,46,47]. This may explain why the relationship between forage and meat was clearer for mutton and horse meat than for beef. Cattle are often kept under more mixed feeding conditions, which can weaken the direct link between local pasture vegetation and the elemental composition of beef. At the same time, these associations should be interpreted carefully, because meat composition is affected by several biological and management-related factors, not by forage alone.

Several limitations should be considered when interpreting the results. First, spatially matched soil–forage–meat sample pairs were not available at the level of individual sampling points. Therefore, the forage–meat correlations describe territorial-level associations rather than direct transfer from a specific pasture to a specific animal product. Second, the study did not include source-tracing analysis, so elevated element concentrations cannot be assigned to individual industrial facilities or emission sources. Third, the food safety assessment was based on comparison with regulatory limits, while dietary exposure and human health risk were not calculated. These limitations do not reduce the value of the observed territorial patterns, but they require cautious interpretation of transfer pathways and contamination sources. Future studies should include spatially matched soil, forage, and animal-product samples, together with source-tracing approaches, to better distinguish direct transfer pathways from broader territorial contamination patterns.

These findings show that monitoring in industrial territories should not be limited to soil analysis alone. Soil data are important for identifying contamination accumulation, but forage and meat analysis are needed to understand whether elements enter the agricultural food chain. This is especially relevant for territories where livestock production depends on local pasture vegetation. An integrated soil–forage–meat approach can help identify elements with higher transfer potential, territories with greater contamination pressure, and animal products that require closer food safety control. In the studied areas, special attention should be given to Cd, Hg, and As in meat products and to Cd, Cu, Pb, and Zn in the soil–forage pathway.

## 5. Conclusions

Industrial impact in Central Kazakhstan was associated with clear territorial differences in the distribution of toxic and essential elements within the soil–forage–meat system. Soil acted as the main accumulation matrix, while forage vegetation retained the territorial pattern at lower concentration levels. The strongest spatial contrast was observed in Temirtau and especially in Zhezkazgan, where elevated element levels reflected a higher local environmental burden. Meat products generally contained lower concentrations than soil and forage, indicating limited transfer of many elements into animal tissues. However, exceedances of regulatory limits for cadmium, mercury, and arsenic show that food safety concerns remain relevant, particularly for horse meat and beef.

The soil-to-forage transfer assessment identified cadmium, copper, lead, and zinc as the elements with the highest relative transfer into forage vegetation. Cluster analysis and principal component analysis confirmed the territorial structure of contamination and separated Zhezkazgan as the most distinct area by elemental profile. Forage–meat correlations were stronger in mutton and horse meat than in beef. This suggests that animal type and feeding conditions may affect how territorial element patterns are reflected in meat. However, these associations should be interpreted at the territorial level. By integrating soil, forage vegetation, and meat products within a single framework, this study provides new insight into the behavior and propagation of trace elements across interconnected components of the regional soil–plant–animal system. Overall, the integrated assessment of soil, forage vegetation, and meat products provides a useful framework for interpreting element distribution and potential transfer pathways within the soil–plant–animal system. This approach helps identify priority elements, affected territories, and meat products that require targeted monitoring in industrial regions, thereby supporting environmental risk assessment and food safety management.

## Figures and Tables

**Table 1:** Average concentrations of the studied elements in soil, forage vegetation, and meat products from the studied territories, mg/kg.

Matrix	Area	n	Mean ± SD											
			As	Cd	Be	V	Cr	Mn	Co	Ni	Cu	Zn	Hg	Pb
Soil	Karaganda	25	5.62 ± 2.01	0.3 ± 0.66	0.72 ± 0.25	60.52 ± 17.38	37.85 ± 12.37	721.87 ± 184.9	7.73 ± 2.33	23.48 ± 10.74	41.09 ± 91	66.45 ± 75.48	0.04 ± 0.07	14.38 ± 17.63
Soil	Saran	44	5.7 ± 5.91	0.14 ± 0.08	0.58 ± 0.3	41.1 ± 23.88	26.63 ± 18.52	480.94 ± 251.58	6.99 ± 5.05	15.36 ± 8.44	13.68 ± 8.32	27.67 ± 11.9	0.04 ± 0.07	9.93 ± 5.65
Soil	Shakhtinsk	66	5.51 ± 2.42	0.14 ± 0.09	0.69 ± 0.31	46.11 ± 18.36	32.11 ± 14.38	589.79 ± 357.23	6.2 ± 3.27	19.6 ± 8.88	13.79 ± 6.81	34.92 ± 13.86	0.02 ± 0.04	9.71 ± 4.59
Soil	Temirtau	28	9.16 ± 3.38	0.5 ± 0.34	0.84 ± 0.26	58.41 ± 17.54	43.99 ± 11.98	844.89 ± 361.6	9.16 ± 6.37	24.3 ± 6.96	38.55 ± 18.65	112.67 ± 85.28	0.02 ± 0.01	32.11 ± 28.09
Soil	Zhezkazgan	40	12.37 ± 5.4	1.33 ± 1.61	1.04 ± 0.4	68.05 ± 23.69	50.72 ± 21.22	859.94 ± 461.47	8.38 ± 4.25	28.99 ± 13.48	271.93 ± 280.64	119.7 ± 136.26	0.03 ± 0.04	73.91 ± 51.9
Forage	Karaganda	25	0.34 ± 0.19	0.3 ± 0.17	0.02 ± 0.02	1.51 ± 0.92	2.49 ± 1.97	91.15 ± 40.69	0.31 ± 0.19	1.76 ± 1.88	6.82 ± 3.71	37.53 ± 20.58	0.01 ± 0.01	3.25 ± 1.93
Forage	Saran	44	0.29 ± 0.2	0.16 ± 0.15	0.02 ± 0.02	1.13 ± 0.91	2.09 ± 1.01	96.16 ± 43.6	0.27 ± 0.25	1.23 ± 1.27	6.01 ± 4.63	29.15 ± 16.85	0.03 ± 0.04	0.96 ± 0.54
Forage	Shakhtinsk	66	0.65 ± 0.43	0.28 ± 0.17	0.06 ± 0.05	3.11 ± 2.74	3.56 ± 2.28	151.02 ± 77.31	0.61 ± 0.46	2.78 ± 1.87	10.54 ± 28.81	31.26 ± 13.16	0.03 ± 0.05	1.85 ± 2.04
Forage	Temirtau	28	1.03 ± 0.52	0.32 ± 0.27	0.08 ± 0.06	3.9 ± 2.61	3.96 ± 2.06	137.7 ± 47.24	0.72 ± 0.49	2.95 ± 1.9	29.73 ± 25.41	32.72 ± 10.82	0.03 ± 0.04	3.26 ± 1.2
Forage	Zhezkazgan	40	5.42 ± 4.33	4.72 ± 5.94	0.09 ± 0.05	3.74 ± 2.14	4.29 ± 1.89	105.2 ± 54.59	0.77 ± 0.45	3.27 ± 1.94	326.05 ± 335.66	89.13 ± 85.71	0.06 ± 0.05	166.56 ± 157.33
Meat	Karaganda	72	0 ± 0	0 ± 0.01	0 ± 0	0.01 ± 0.01	3.11 ± 4.36	0.24 ± 0.3	0.01 ± 0.03	1.18 ± 2.53	0.84 ± 0.49	94.79 ± 70.48	0 ± 0.02	0.03 ± 0.07
Meat	Saran	66	0 ± 0	0 ± 0.01	0 ± 0	0.01 ± 0.02	4.98 ± 5.71	0.35 ± 0.34	0.02 ± 0.03	2.06 ± 2.6	0.91 ± 0.45	76.96 ± 63.98	0 ± 0.01	0.02 ± 0.04
Meat	Shakhtinsk	37	0 ± 0.01	0.01 ± 0.01	0 ± 0	0.02 ± 0.04	8.3 ± 6.11	0.5 ± 0.36	0.04 ± 0.03	3.49 ± 2.82	0.98 ± 0.53	70.26 ± 42.31	0 ± 0.01	0.03 ± 0.05
Meat	Temirtau	63	0.01 ± 0.02	0.01 ± 0.03	0 ± 0	0.01 ± 0.01	3.66 ± 4.98	0.28 ± 0.46	0.02 ± 0.03	1.38 ± 2.31	0.89 ± 0.44	83.39 ± 61.81	0 ± 0.02	0.03 ± 0.07
Meat	Zhezkazgan	41	0.02 ± 0.01	0.03 ± 0.05	0 ± 0	0.04 ± 0.01	9.52 ± 2.33	0.88 ± 0.26	0.06 ± 0.01	5.03 ± 2.09	1.28 ± 0.52	92.98 ± 48.2	0.01 ± 0.01	0.06 ± 0.06

**Table 2:** Number and percentage of meat samples exceeding the maximum permissible concentrations of Cd, Hg, and As according to the Eurasian Economic Union (EAEU) Technical Regulations.

Sample Type	n	Cd n (%)	Hg n (%)	As n (%)
Beef	193	5 (2.6%)	3 (1.5%)	1 (0.5%)
Horsemeat	44	9 (20.4%)	0 (0.0%)	0 (0.0%)
Mutton	42	1 (2.4%)	4 (9.5%)	0 (0.0%)
Total	279	15 (5.4%)	7 (2.5%)	1 (0.3%)

Note: Maximum permissible concentrations established by the Eurasian Economic Union (EAEU) Technical Regulations were 0.05 mg/kg for Cd, 0.03 mg/kg for Hg, and 0.10 mg/kg for As.

## Data Availability

The original contributions presented in this study are included in the article and Supplementary Materials. Further inquiries can be directed to the corresponding author.

## Supplementary Materials

The following supporting information can be downloaded at https://www.mdpi.com/article/10.3390/biology15131011/s1. All supplementary information is included in the Supplementary File. Table S1: Descriptive statistics (mean, median, standard deviation, minimum, maximum, and sample size) of element concentrations in soil, forage vegetation, and meat samples from the studied territories; Material S2: Explanation of Principal Component Analysis (PCA); Material S3: Spearman’s Rank Correlation Analysis.

## Abbreviations

The following abbreviations are used in this manuscript:

ICP-MS	Inductively coupled plasma mass spectrometry
PCA	Principal component analysis
TF	Transfer coefficient
