# Supplementary material for: Dynamics of Toxic and Essential Element Transfer in Soil–Plant–Animal Systems Under Industrial Contamination
Source: Biology (Basel). 2026 Jun 25;15(13):1011. doi: 10.3390/biology15131011 (PMC13359659; doi:10.3390/biology15131011)
Supplement: Supplementary file 1 [file biology-15-01011-s001.zip › biology-4387133-supplementary.pdf]

Supplementary Table S1

Descriptive statistics (mean, median, standard deviation, minimum, maximum, and sample size) of element concentrations in soil, forage vegetation, and meat samples from the studied territories.

Matrix	Area	Element	Mean	Median	SD	Min	Max	n
Soil	Karaganda	As	5,62	5,39	2,01	2,33	9,84	25
Soil	Karaganda	Be	0,72	0,66	0,25	0,42	1,6	25
Soil	Karaganda	Cd	0,3	0,16	0,66	0,07	3,43	25
Soil	Karaganda	Co	7,73	6,95	2,33	3,41	12,79	25
Soil	Karaganda	Cr	37,85	35,71	12,37	12,64	74,05	25
Soil	Karaganda	Cu	41,09	18,4	91	11	468,94	25
Soil	Karaganda	Hg	0,04	0,02	0,07	0	0,35	25
Soil	Karaganda	Mn	721,87	743,93	184,9	418,77	1134,76	25
Soil	Karaganda	Ni	23,48	20,08	10,74	10,84	60,95	25
Soil	Karaganda	Pb	14,38	10,69	17,63	5,84	95,29	25
Soil	Karaganda	V	60,52	59	17,38	32,43	113,56	25
Soil	Karaganda	Zn	66,45	50,02	75,48	32,99	420,13	25
Soil	Saran	As	5,7	4,78	5,91	2,01	40,89	44
Soil	Saran	Be	0,58	0,57	0,3	0,22	1,87	44
Soil	Saran	Cd	0,14	0,12	0,08	0,04	0,38	44
Soil	Saran	Co	6,99	5,4	5,05	1,33	28,79	44
Soil	Saran	Cr	26,63	23,03	18,52	5,63	93,43	44
Soil	Saran	Cu	13,68	11,58	8,32	4,23	42,35	44
Soil	Saran	Hg	0,04	0,02	0,07	0	0,34	44
Soil	Saran	Mn	480,94	477,04	251,58	82,18	1427,48	44
Soil	Saran	Ni	15,36	14,41	8,44	4,54	42,77	44
Soil	Saran	Pb	9,93	9,15	5,65	3,33	35,12	44
Soil	Saran	V	41,1	38,89	23,88	9,46	146,93	44
Soil	Saran	Zn	27,67	27,9	11,9	8,82	55,76	44
Soil	Shakhtinsk	As	5,51	4,77	2,42	2,13	15,72	66
Soil	Shakhtinsk	Be	0,69	0,61	0,31	0,27	1,79	66
Soil	Shakhtinsk	Cd	0,14	0,12	0,09	0,01	0,58	66
Soil	Shakhtinsk	Co	6,2	5,2	3,27	2,32	19,43	66
Soil	Shakhtinsk	Cr	32,11	29,8	14,38	8,75	80,4	66
Soil	Shakhtinsk	Cu	13,79	11,84	6,81	3,95	33,7	66
Soil	Shakhtinsk	Hg	0,02	0,01	0,04	0	0,28	66
Soil	Shakhtinsk	Mn	589,79	488,1	357,23	227,79	2675,46	66
Soil	Shakhtinsk	Ni	19,6	17,06	8,88	7,92	56,66	66
Soil	Shakhtinsk	Pb	9,71	7,76	4,59	3,26	25,6	66
Soil	Shakhtinsk	V	46,11	40,89	18,36	12,36	94,81	66
Soil	Shakhtinsk	Zn	34,92	30,65	13,86	14,17	96,17	66
Soil	Temirtau	As	9,16	8,71	3,38	2,61	19,64	28
Soil	Temirtau	Be	0,84	0,81	0,26	0,25	1,41	28

Soil	Temirtau	Cd	0,5	0,45	0,34	0,18	1,53	28
Soil	Temirtau	Co	9,16	7,81	6,37	1,78	38,17	28
Soil	Temirtau	Cr	43,99	43,38	11,98	22,44	73,47	28
Soil	Temirtau	Cu	38,55	34,43	18,65	18,63	99,86	28
Soil	Temirtau	Hg	0,02	0,02	0,01	0,01	0,04	28
Soil	Temirtau	Mn	844,89	751,55	361,6	255,72	1661,29	28
Soil	Temirtau	Ni	24,3	24,35	6,96	11,2	42,41	28
Soil	Temirtau	Pb	32,11	24,32	28,09	5,48	156,01	28
Soil	Temirtau	V	58,41	58,14	17,54	15,18	98,27	28
Soil	Temirtau	Zn	112,67	76,94	85,28	47,83	425,12	28
Soil	Zhezkazgan	As	12,37	12,01	5,4	3,52	24,79	40
Soil	Zhezkazgan	Be	1,04	1,18	0,4	0,27	1,63	40
Soil	Zhezkazgan	Cd	1,33	0,75	1,61	0,09	6,67	40
Soil	Zhezkazgan	Co	8,38	7,75	4,25	1,94	24,03	40
Soil	Zhezkazgan	Cr	50,72	59,17	21,22	8,99	79,96	40
Soil	Zhezkazgan	Cu	271,93	158,34	280,64	9,87	1264,82	40
Soil	Zhezkazgan	Hg	0,03	0,02	0,04	0	0,18	40
Soil	Zhezkazgan	Mn	859,94	762,61	461,47	170,74	2588,86	40
Soil	Zhezkazgan	Ni	28,99	31,9	13,48	5,99	59,14	40
Soil	Zhezkazgan	Pb	73,91	50,84	51,9	9,55	218,93	40
Soil	Zhezkazgan	V	68,05	73,14	23,69	16,2	98,59	40
Soil	Zhezkazgan	Zn	119,7	81,58	136,26	15,94	562,98	40
Forage	Karaganda	As	0,34	0,32	0,19	0,07	0,76	25
Forage	Karaganda	Be	0,02	0,02	0,02	0	0,06	25
Forage	Karaganda	Cd	0,3	0,22	0,17	0,08	0,87	25
Forage	Karaganda	Co	0,31	0,28	0,19	0,07	0,69	25
Forage	Karaganda	Cr	2,49	1,7	1,97	0,72	8,36	25
Forage	Karaganda	Cu	6,82	6,57	3,71	1,69	17,77	25
Forage	Karaganda	Hg	0,01	0,01	0,01	0	0,03	25
Forage	Karaganda	Mn	91,15	90,2	40,69	19,94	190,05	25
Forage	Karaganda	Ni	1,76	1,2	1,88	0	6,19	25
Forage	Karaganda	Pb	3,25	2,55	1,93	1,01	8,47	25
Forage	Karaganda	V	1,51	1,37	0,92	0,32	3,53	25
Forage	Karaganda	Zn	37,53	32,35	20,58	8,79	94,61	25
Forage	Saran	As	0,29	0,22	0,2	0,08	1,08	44
Forage	Saran	Be	0,02	0,01	0,02	0	0,08	44
Forage	Saran	Cd	0,16	0,12	0,15	0,01	0,66	44
Forage	Saran	Co	0,27	0,17	0,25	0,05	1,15	44
Forage	Saran	Cr	2,09	1,8	1,01	0,53	6,72	44
Forage	Saran	Cu	6,01	4,34	4,63	1,73	23,36	44
Forage	Saran	Hg	0,03	0,01	0,04	0	0,24	44
Forage	Saran	Mn	96,16	85,42	43,6	34,31	256,84	44
Forage	Saran	Ni	1,23	0,95	1,27	0	6,71	44
Forage	Saran	Pb	0,96	0,87	0,54	0,21	2,85	44

Forage	Saran	V	1,13	0,72	0,91	0,24	4,21	44
Forage	Saran	Zn	29,15	22,94	16,85	6,28	73,77	44
Forage	Shakhtinsk	As	0,65	0,55	0,43	0,12	2,21	66
Forage	Shakhtinsk	Be	0,06	0,05	0,05	0	0,26	66
Forage	Shakhtinsk	Cd	0,28	0,24	0,17	0,02	1,07	66
Forage	Shakhtinsk	Co	0,61	0,5	0,46	0,08	2,32	66
Forage	Shakhtinsk	Cr	3,56	3,13	2,28	0,88	13,03	66
Forage	Shakhtinsk	Cu	10,54	6,74	28,81	1,26	239,65	66
Forage	Shakhtinsk	Hg	0,03	0,01	0,05	0	0,28	66
Forage	Shakhtinsk	Mn	151,02	146,17	77,31	41,12	435,87	66
Forage	Shakhtinsk	Ni	2,78	2,39	1,87	0,47	8,99	66
Forage	Shakhtinsk	Pb	1,85	1,46	2,04	0,34	16,17	66
Forage	Shakhtinsk	V	3,11	2,56	2,74	0,32	18,36	66
Forage	Shakhtinsk	Zn	31,26	29,61	13,16	9,82	74,71	66
Forage	Temirtau	As	1,03	1,06	0,52	0,17	2,18	28
Forage	Temirtau	Be	0,08	0,07	0,06	0,01	0,25	28
Forage	Temirtau	Cd	0,32	0,27	0,27	0,05	1,37	28
Forage	Temirtau	Co	0,72	0,6	0,49	0,1	2,11	28
Forage	Temirtau	Cr	3,96	3,63	2,06	1,38	9,46	28
Forage	Temirtau	Cu	29,73	15,81	25,41	6,56	84,65	28
Forage	Temirtau	Hg	0,03	0,02	0,04	0,01	0,2	28
Forage	Temirtau	Mn	137,7	134,72	47,24	67,17	295,18	28
Forage	Temirtau	Ni	2,95	2,51	1,9	0,39	8,34	28
Forage	Temirtau	Pb	3,26	3,37	1,2	1,06	6,52	28
Forage	Temirtau	V	3,9	3,23	2,61	0,74	11,63	28
Forage	Temirtau	Zn	32,72	30,09	10,82	11,26	56,07	28
Forage	Zhezkazgan	As	5,42	4,28	4,33	0,84	16,7	40
Forage	Zhezkazgan	Be	0,09	0,08	0,05	0,02	0,21	40
Forage	Zhezkazgan	Cd	4,72	3,36	5,94	0,19	32,62	40
Forage	Zhezkazgan	Co	0,77	0,65	0,45	0,19	1,93	40
Forage	Zhezkazgan	Cr	4,29	3,98	1,89	1,36	8,96	40
Forage	Zhezkazgan	Cu	326,05	257,82	335,66	15,08	1595,07	40
Forage	Zhezkazgan	Hg	0,06	0,06	0,05	0	0,19	40
Forage	Zhezkazgan	Mn	105,2	95,05	54,59	34,23	264,67	40
Forage	Zhezkazgan	Ni	3,27	2,98	1,94	0,7	8,98	40
Forage	Zhezkazgan	Pb	166,56	144,82	157,33	8,09	694,54	40
Forage	Zhezkazgan	V	3,74	3,36	2,14	0,8	8,8	40
Forage	Zhezkazgan	Zn	89,13	57,05	85,71	15,74	438,53	40
Meat	Karaganda	As	0	0	0	0	0,02	72
Meat	Karaganda	Be	0	0	0	0	0	72
Meat	Karaganda	Cd	0	0	0,01	0	0,07	72
Meat	Karaganda	Co	0,01	0	0,03	0	0,1	72
Meat	Karaganda	Cr	3,11	1,11	4,36	0	18,57	72
Meat	Karaganda	Cu	0,84	0,72	0,49	0,09	2,65	72

Meat	Karaganda	Hg	0	0	0,02	0	0,15	72
Meat	Karaganda	Mn	0,24	0,12	0,3	0	1,36	72
Meat	Karaganda	Ni	1,18	0,07	2,53	0	14,94	72
Meat	Karaganda	Pb	0,03	0	0,07	0	0,35	72
Meat	Karaganda	V	0,01	0	0,01	0	0,04	72
Meat	Karaganda	Zn	94,79	69,41	70,48	7,35	334,04	72
Meat	Saran	As	0	0	0	0	0,02	66
Meat	Saran	Be	0	0	0	0	0	66
Meat	Saran	Cd	0	0	0,01	0	0,1	66
Meat	Saran	Co	0,02	0	0,03	0	0,09	66
Meat	Saran	Cr	4,98	1,23	5,71	0	17,66	66
Meat	Saran	Cu	0,91	0,8	0,45	0,23	2,22	66
Meat	Saran	Hg	0	0	0,01	0	0,04	66
Meat	Saran	Mn	0,35	0,16	0,34	0	1,45	66
Meat	Saran	Ni	2,06	0,3	2,6	0	7,64	66
Meat	Saran	Pb	0,02	0	0,04	0	0,22	66
Meat	Saran	V	0,01	0	0,02	0	0,06	66
Meat	Saran	Zn	76,96	65,98	63,98	7,64	363,48	66
Meat	Shakhtinsk	As	0	0	0,01	0	0,03	37
Meat	Shakhtinsk	Be	0	0	0	0	0	37
Meat	Shakhtinsk	Cd	0,01	0	0,01	0	0,08	37
Meat	Shakhtinsk	Co	0,04	0,05	0,03	0	0,11	37
Meat	Shakhtinsk	Cr	8,3	10,93	6,11	0	16,43	37
Meat	Shakhtinsk	Cu	0,98	0,84	0,53	0,02	2,59	37
Meat	Shakhtinsk	Hg	0	0	0,01	0	0,02	37
Meat	Shakhtinsk	Mn	0,5	0,54	0,36	0	1,49	37
Meat	Shakhtinsk	Ni	3,49	4,81	2,82	0	7,2	37
Meat	Shakhtinsk	Pb	0,03	0,01	0,05	0	0,19	37
Meat	Shakhtinsk	V	0,02	0,02	0,04	0	0,26	37
Meat	Shakhtinsk	Zn	70,26	71,63	42,31	7,19	201,31	37
Meat	Temirtau	As	0,01	0	0,02	0	0,14	63
Meat	Temirtau	Be	0	0	0	0	0	63
Meat	Temirtau	Cd	0,01	0	0,03	0	0,22	63
Meat	Temirtau	Co	0,02	0	0,03	0	0,09	63
Meat	Temirtau	Cr	3,66	1,15	4,98	0	17	63
Meat	Temirtau	Cu	0,89	0,75	0,44	0,3	2,38	63
Meat	Temirtau	Hg	0	0	0,02	0	0,14	63
Meat	Temirtau	Mn	0,28	0,14	0,46	0	3,27	63
Meat	Temirtau	Ni	1,38	0,1	2,31	0	7,13	63
Meat	Temirtau	Pb	0,03	0	0,07	0	0,39	63
Meat	Temirtau	V	0,01	0	0,01	0	0,06	63
Meat	Temirtau	Zn	83,39	66,43	61,81	6,77	368,93	63
Meat	Zhezkazgan	As	0,02	0,02	0,01	0,01	0,03	41
Meat	Zhezkazgan	Be	0	0	0	0	0	41

Meat	Zhezkazgan	Cd	0,03	0	0,05	0	0,26	41
Meat	Zhezkazgan	Co	0,06	0,06	0,01	0,04	0,11	41
Meat	Zhezkazgan	Cr	9,52	8,47	2,33	6,86	15,39	41
Meat	Zhezkazgan	Cu	1,28	1,11	0,52	0,56	2,95	41
Meat	Zhezkazgan	Hg	0,01	0	0,01	0	0,06	41
Meat	Zhezkazgan	Mn	0,88	0,84	0,26	0,56	1,63	41
Meat	Zhezkazgan	Ni	5,03	4,03	2,09	3,14	10,41	41
Meat	Zhezkazgan	Pb	0,06	0,04	0,06	0	0,23	41
Meat	Zhezkazgan	V	0,04	0,04	0,01	0,02	0,05	41
Meat	Zhezkazgan	Zn	92,98	86,02	48,2	24,93	209,64	41

Supplementary material S2

Explanation of Principal Component Analysis (PCA)

Principal component analysis (PCA) was used to identify patterns in the distribution of elements in soil, forage, and meat samples. Prior to analysis, the data were centered and standardized to eliminate differences in measurement scales among the studied elements.

Standardization was performed according to the following equation:

$$Zij = (xij - \bar{x}j) / sj$$

where xij is the original concentration of an element, $\bar{x}j$ is the mean concentration of the element, sj is the standard deviation, and Zij is the standardized value.

The principal components were calculated as linear combinations of the standardized variables:

$$PCk = a1kZ1 + a2kZ2 + ... + apkZp$$

where PCk is the k-th principal component, aik are the loading coefficients of the corresponding variables, and Zi are the standardized values of the analyzed elements.

In the PCA plots, PC1 and PC2 represent the first and second principal components, respectively. PC1 explains the largest proportion of the total variance in the dataset, whereas PC2 explains the largest proportion of the remaining variance not accounted for by PC1.

In the loading plots, Dim1 and Dim2 correspond to the first (PC1) and second (PC2) principal components, respectively. The direction and length of the vectors indicate the contribution of individual elements to the principal components and the relationships among variables.

The percentages shown in parentheses on the PC1 and PC2 axes indicate the proportion of the total variance explained by each principal component. The total variance of all variables included in the analysis was considered as 100%.

Supplementary Material S3.

Spearman’s Rank Correlation Analysis

Spearman’s rank correlation coefficient (ρ) was used to evaluate the agreement between the elemental composition of forage and meat samples. The coefficient was calculated based on the ranks of the compared variables according to the following equation:

$$\rho = 1 - [6 \times \Sigma(d_i^2)] / [n(n^2 - 1)]$$

where:

ρ is Spearman’s rank correlation coefficient;

d_i is the difference between the ranks of the i-th element in the two compared datasets;

n is the number of observations.

The coefficient ranges from −1 to +1. A value of $\rho = +1$ indicates a perfect positive monotonic relationship, whereas $\rho = -1$ indicates a perfect negative monotonic relationship. A value of $\rho = 0$ indicates the absence of a monotonic association between variables.

In this study, Spearman’s correlation coefficients were calculated separately for each territory and each meat type using the mean concentrations of the 12 studied elements in forage and meat samples.
